# Prevalence and Impact of Violence Against Healthcare Workers in Brazilian Emergency Departments: A National Survey

**DOI:** 10.5811/westjem.45138

**Published:** 2025-10-17

**Authors:** Julia M. Dorn de Carvalho, Sarayna S. McGuire, Lucas L. R. Oliveira, Fernanda Bellolio, Otávio T. Ranzani, Bruno A.M Pinheiro Besen, Helio Penna Guimarães, Maria Camila Lunardi, Aidan F. Mullan, Ludhmila A. Hajjar, Ian Ward A. Maia

**Affiliations:** * Hospital das Clínicas da Faculdade de Medicina da Universidade de São Paulo, Department of Emergency Medicine, São Paulo, Brazil; † Mayo Clinic, Department of Emergency Medicine, Mayo Clinic, Rochester, Minnesota; ‡ ISGlobal, Barcelona, Spain; § Institut de Recerca Sant Pau (IR SANT PAU), Barcelona, Spain; ¶ IDOR Education and Research Institute, São Paulo, Brazil; || Hospital das Clínicas da Faculdade de Medicina da Universidade de São Paulo, Heart Institute, Faculty Medicine, Pulmonary Division, São Paulo, Brazil; # Faculdade de Medicina, Universidade de São Paulo, Internal Medicine Department, Medical Sciences Postgraduate Programme, São Paulo, Brazil; ** Mayo Clinic, Department of Quantitative Health Sciences, Rochester, Minnesota

## Abstract

**Introduction:**

Workplace violence (WPV) is a significant occupational hazard in healthcare, with emergency departments (EDs) recognized as high-risk environments. Although globally significant, data from Latin America remain scarce. In this study we aimed to evaluate the prevalence and effects of WPV on healthcare workers in Brazilian EDs.

**Methods:**

We conducted a cross-sectional survey of healthcare workers in Brazilian EDs. Respondents indicated verbal and physical violence experienced within the preceding six months, along with associated psychological and occupational impacts. Univariable models identified significant associated factors, followed by multivariable models to determine independent associated factors of WPV. We reported results as adjusted odds ratios (aOR) with 95% confidence intervals. Statistical analyses were performed in R v4.4.1, and significance was defined as P < .05.

**Results:**

The response rate was 19.1% (1,255/6,570), Of those responses, 61.3% (769/1,255) met the inclusion criteria and were included in the analysis. Of all respondents, 84.0% were physicians. Respondents indicated 79.6% (612/769) occurrence of WPV, including verbal abuse (79.5%) and physical assault (12.1%). Physical assaults against co-workers were witnessed by 40.3% of respondents. Perpetrators included visitors (85.3%), patients (80.7%), and co-workers (35.8%). The absence of institutional preventive measures was associated with increased WPV (aOR, 2.47; 95% CI, 1.71–3.57; P < .001), while the presence of security staff reduced WPV (aOR, 0.61; 95% CI, 0.42–0.89; P = .01). Indicated impact included post-traumatic stress symptoms (88.4%), considering leaving their job (49.5%), impaired workplace performance (75.2%), and time off work (10%), including 11.5% permanently leaving.

**Conclusion:**

Workplace violence is highly prevalent in Brazilian EDs, with substantial psychological and occupational consequences. The absence of protocols or preventive measures may increase WPV risk, emphasizing the urgent need for public policies to protect healthcare workers in emergency settings.

## INTRODUCTION

Workplace violence (WPV) has emerged as a critical occupational hazard in healthcare since its initial recognition in the 1990s.^1^–^3^ The Joint Commission report defines WPV as “any act or threat that occurs in the workplace, including verbal, nonverbal, written, or physical assaults, as well as threats, intimidation, harassment, or humiliating words or actions”.^4^ A systematic review including studies from Asia, Europe, and North America reported that 61.9% of healthcare workers experience WPV, with 42.5% indicating verbal abuse and 24.4% encountering physical assault.^5^

The emergency department (ED) is a high-risk environment for violence, driven by factors such as crowding, staff shortages, off-hours work, and the presence of patients with substance use disorder, acute intoxication, behavioral health conditions, delirium, or altered mental status.^6^–^8^ Recent surveys conducted in ED of the United States and a systematic review found that 71.5% to 75.0% of healthcare workers indicated being victims of verbal abuse, while physical assault was reported by 18–30.8%.^7^,^9^–^11^ Workplace violence significantly impacts staff, increasing the risk of burnout and post-traumatic stress disorder (PTSD) and decreasing job satisfaction, all of which threaten staff retention and lead to high staff turnover.^12^

Furthermore, WPV impacts patient care and has significant effects on the healthcare system, with increased financial costs due to healthcare needs for staff injuries and absenteeism following a violent event.^6^ These findings have led national healthcare organizations to implement preventive measures, including legislative reforms, institutional protocols, and increased criminal penalties for WPV.^13^ Although single-center studies in Brazil show WPV rates similar to those reported in other countries, a national assessment is lacking, limiting awareness and establishment. of effective interventions.^14^,^15^

Therefore, we conducted a national cross-sectional survey to determine the prevalence of WPV in EDs across Brazil and to assess how WPV affects healthcare workers.

## METHODS

### Study Design and Setting

We conducted a cross-sectional survey of healthcare workers in EDs across Brazil. The survey was administered using the Research Electronic Data Capture (REDCap) platform, hosted at Hospital das Clínicas da Faculdade de Medicina da Universidade de São Paulo.^16^ Informed consent was obtained at the beginning of the survey, with respondents indicating their agreement by responding affirmatively to the initial consent question. Responses were collected anonymously. The study protocol was approved by the institutional review board. This study is reported in accordance with the Checklist for Reporting Results of Internet E-Surveys (CHERRIES) guidelines.^17^

The setting was Brazilian EDs, which is divided into three sectors: the public sector (Unified Health System, SUS); the private sector; and the private health insurance sector.^18^ Public emergency care encompasses prehospital services, community EDs, and hospital facilities. Community EDs, which provide services of intermediate complexity, along with some hospitals, serve as primary access points to emergency care. Meanwhile, other EDs are referral-only centers that primarily treat patients referred from lower complexity healthcare services, along with a smaller proportion of individuals from prehospital care and those seeking care independently. The public sector serves the majority of the population, with 74% of individuals relying on public hospitals for care.^19^ However, EDs in the public sector face significant challenges, including crowding, elevated mortality rates, and staffing shortages.^18^,^20^ Furthermore, emergency medicine (EM). is a relatively new specialty in Brazil, officially recognized only in 2015^21^ and represented just 0.2% of all medical specialties in 2022.^22^

**Population Health Research Capsule**

What do we already know about this issue?

*Workplace violence is a major occupational hazard in emergency departments worldwide, associated with negative psychological impacts.*

What was the research question?

*What is the prevalence and impact of workplace violence on ED healthcare workers across Brazil?*

What was the major finding of the study?

*Workplace violence affected 79.6% of respondents, and the absence of prevention measures was associated with higher odds of exposure (adjusted OR, 1.88; 95% CI, 1.10–3.21; P = .02).*

How does this improve population health?

*This study highlights the need of policies to protect ED staff and ensure care quality under high-risk conditions.*

### Selection of Participants

The target population was Brazilian healthcare staff who had worked in the ED for at least 12 hours a week in the prior six months (March/April to September/October 2024). These healthcare workers included physicians, nurses, nursing technicians, and respiratory therapists, including workers in training. The survey was distributed by email using the distribution list of the Brazilian Association of Emergency Medicine (ABRAMEDE), which includes 6,570 members, primarily consisting of emergency physicians. Four reminders were sent during the collection period. We included in the study only the respondents who consented to the research, completed the survey, and had worked in the ED for at least 12 hours a week in the prior six months. To prevent multiple entries from the same individual, a log file analysis was carried out by the REDCap platform to ensure only one response by the individual link sent to each email.

### Survey Development and Measurements

Survey participation was voluntary, with no incentives provided. Responses were collected via the REDCap platform from September 11–October 14, 2024. The survey (eTable 4, Appendix 1) was adapted from McGuire et al,^6^,^11^ which examined WPV in Midwestern EDs in the United States. To ensure clarity and comprehension, we conducted a pilot study with 150 emergency physicians and EM residents. The Portuguese-language survey was comprised of 76 questions organized into five categories: demographic data, including self-reported sex and race; verbal abuse; physical assault; institutional characteristics; and the impact of violence on staff. Respondents were able to review and change their answers through a “back button,” and all surveys were checked for completeness after submission.

Staff were asked whether they had experienced verbal abuse or physical assault, either personally or as a witness, over the prior six months. The definition of verbal abuse, as outlined by Farrell et al,^23^ was “any form of mistreatment, whether explicit or implied, that causes feelings of devaluation or humiliation through derogatory language, threats, accusations, or disrespectful expressions.”. The definition of physical assault was based on the World Health Organization’s description as “the use of physical force that results in physical, sexual, or psychological harm.”^24^

For those who indicated experiencing verbal abuse, further questions were asked to categorize the type of abuse, including: threatening tone, abusive language, verbal harassment (racial, sexual, gender-based, or other), or other forms of verbal abuse. Regarding physical assault, respondents were asked to specify the nature of the assault, including physical assault with objects, body fluids, physical harm with punching, biting, rough handling, scratching, kicking, shoving/pushing, or hitting, sexual assault, or other forms of physical assault.

For each type of verbal abuse and physical assault, respondents were asked to indicate the frequency of occurrence over the prior six months: none; once; 2–5 times; 5–10 times; or more than 10 times. They were also asked to identify the perpetrator—whether a patient, visitor, or coworker—and specify the frequency of such incidents for each type of perpetrator over the prior six months using the same frequency options. Additionally, staff were questioned about the impact of the violence, such as whether it necessitated taking time off work (days, weeks, or permanently), influenced their consideration of leaving their job, affected their interactions with patients, or led to symptoms of post-traumatic stress.

Respondents were also surveyed regarding their perception of safety in the ED, as well as the availability of institutional WPV preventive measures, such as protocols for managing severe agitation, staff training, security personnel in the ED, and the presence of metal detectors. They were further questioned about their preferred medication for managing severe agitation, with options including ketamine, haloperidol, a benzodiazepine, a combination of haloperidol and a benzodiazepine, a combination of haloperidol and promethazine, propofol, or other medications.

### Outcomes

The primary outcome was the prevalence of WPV, defined as the presence of verbal or physical assault experienced by healthcare workers during their clinical shift. To minimize recall bias, the assessment period was restricted to incidents occurring within the six months prior to the survey. Secondary outcomes included the prevalence of witnessed physical assault, the prevalence of workers temporarily absent from work due to assault, the incidence of post-traumatic stress symptoms, and description of factors associated with verbal and physical assault.

### Analysis

The response rate was defined as the number of people who responded to the survey divided by the number of total potential respondents. We summarized the survey responses using frequency counts and percentages. Demographic characteristics and ED practice features were compared between respondents who indicated experiencing WPV and those who did not, using two-sided chi-square tests or Fisher exact tests, as appropriate. We employed logistic regression to evaluate potential risk factors for WPV, including respondent demographics, ED practice characteristics, and institutional violence prevention measures. The primary outcome was self-reported verbal abuse or physical assault within the prior six months. Based on expert knowledge, we pre-selected variables hypothesised to be risk factors for WPV. These variables were initially assessed using univariable logistic regression.

To account for potential confounding by age and sex, we performed an additional logistic regression model adjusted for these two variables. Finally, variables with significant associations (P < .05) were included in a multivariable logistic regression model to identify independent associated factors of WPV. All statistical analyses were performed using R v4.4.1 (The R Foundation for Statistical Computing, Vienna, Austria). Statistical significance was defined as a two-sided *P*-value of < .05. We calculated nonresponse bias by wave analysis, using survey question 49 “How safe do you feel in the Emergency Department?” as a five-point Likert scale (1 - extremely safe and 5 - not at all safe).^25^

## RESULTS

Of the 6,570 emails sent, 1,255 surveys were returned, with a response rate of 19.1% (1,255/6,570). Of those, 769/6,570 (11.7%) were included in the analysis.^25^ We excluded 391 surveys (31.1%) with incomplete responses, defined as those with at least two unanswered questions; 91 respondents (7.2%) with fewer than six months of ED experience; and four surveys that lacked consent (0.3%) (eFigure 1, Appendix 1). The survey completion rate, calculated as the percentage of respondents who fully completed the survey among those who initially agreed to respond, was 71.8%.^17^ The nonresponse bias was 0.048, with a proportion of nonrespondents of 5,315/6,570, mean true respondents of 3.408 from 115 responses, and mean proxy nonrespondents of 3.467 from 75 responses.^25^

The majority of respondents were physicians (n = 646; 84.0%), of whom 80.5% were attending physicians and 19.5% were resident physicians, followed by 62 who were nurses (8.1%) and 28 nursing technicians (3.6%). Respondent demographic characteristics are summarized in Table 1. The median age of respondents was 32 years, (interquartile range 28–39 years), with most identifying as cisgender women (n = 437; 56.8%) followed by cisgender men (n = 318; 41.4%). The majority identified as heterosexual (n = 642; 83.5%) and White (n = 590; 76.7%). The primary region of practice was the Southeast (n = 371; 48.2%), followed by the South (n = 205; 26.7%), with a minority from the Northeast (n = 120; 15.6%), West (n = 51; 6.6%) and North (n = 22; 2.9%) region.

Most respondents were employed in public hospitals (n = 654; 85.1%), with the majority working in facilities located in capital cities (n = 369; 48.0%) or community EDs (n = 234; 30.4%). Work experience was predominantly more than five years (n = 353; 45.9%) or between 1–5 years (n = 329; 42.8%).

### Prevalence of Workplace Violence

**Figure 1:**
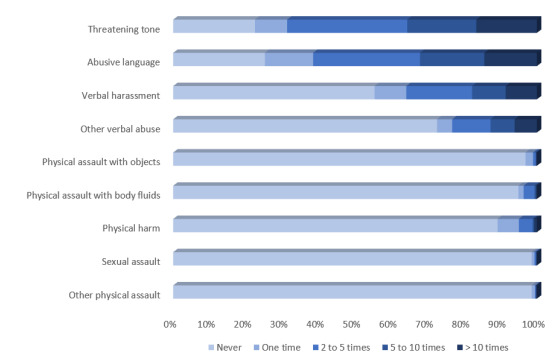
Frequency of each type of workplace violence experienced by Brazilian emergency department staff in the prior six months.

A total of 612 respondents (79.6%) indicated experiencing some form of violence in the preceding six months, including, 611 (79.5%) who had experienced verbal abuse and 93 (12.1%) who had experienced physical assault. Additionally, 310 (40.3%) respondents witnessed physical assault against a co-worker. The frequency of each type of violence is summarized in Table 2. Among those indicating verbal abuse, 596 (97.5%) experienced threatening tone of voice, 575 (94.1%) abusive language, and 343 (56.1%) verbal harassment. Furthermore, among those indicating physical assault, 83 (89.2%) experienced physical harm, 39 (41.9%) were assaulted with body fluids, 24 (25.8%) were assaulted with objects, and 11 (11.8%) indicated sexual assault (Figure 1). Perpetrators were reported as visitors (n = 522; 85.3%), followed by patients (n = 494; 80.7%) and co-workers (n = 275; 35.8%). In most cases, the perpetrator was not perceived to be intoxicated (n = 494; 80.7%) or experiencing altered mental status (n = 485; 79.2%) (Table 2).

### Factors Associated with Workplace Violence

In the multivariable model, the absence of preventive measures (question 63, eTable 4, Appendix 1) in the workplace was associated with an increased risk of WPV (OR, 1.88; 95% CI 1.10–3.21; *P* = .02). In the univariable analysis, the presence of an institutional protocol for managing severe agitation was associated with a reduced risk of WPV (OR, 0.46; 95% CI 0.31–0.70; *P* <.001; Table 3). However, no significant difference was observed in the multivariable analysis (OR, 0.65; 95% CI .39–1.08; *P* = .09). Public EDs were associated with a higher risk of WPV compared to private and referral-only EDs (OR, 1.79; 95% CI 1.24–2.60; *P* = <.001). However, this difference was not significant in the multivariable analysis (OR, 1.41; 95% CI 0.83–2.40; *P* = .20).

Increasing staff age was associated with a reduced risk of WPV (OR, 0.72; 95% CI 0.61–0.84; *P* < .001). There was no significant difference in the multivariable analysis between more than five years of staff experience compared with less than one year (OR, 1.06; 95% CI 0.35–3.22; *P* = .91) and one to five years of experience (OR, 0.84; 95% CI 0.43–1.64; *P* = .61). No significant differences in risk of WPV were observed by staff sex or gender (OR, 1.32; 95% CI 0.92–1.88; *P* = .13), staff sexual orientation (OR, 1.39; 95% CI, 0.82–2.35; *P* = .22), or self-reported non-White skin color (OR, 0.86; 95% CI 0.57–1.29; *P* = .47). No significant differences were found when comparing attending physicians with nurses (OR, 1.25; 95% CI 0.55–2.86; *P* = .59) or residents (OR, 0.79; 95% CI 0.36–1.71; *P* = .54) (Table 3).

### Impact of Workplace Violence and Availability of Institutional Resources

The majority of staff respondents indicated feeling unsafe in the ED (n = 697; 90.6%; eTable 2, Appendix), with only 72 (9.4%) respondents feeling extremely or very safe in their workplace. Among all respondents, 496 (64.5%) indicated that their institution lacked preventive measures. Only 103 (13.4%) respondents reported having an institutional protocol for severe agitation, 195 (25.4%) referenced the presence of security staff, 57 (7.4%) reported available staff training, and none indicated the availability of metal detectors at their ED (eTable 3, Appendix).

Additionally, 469 (61.0%) respondents indicated security staff were unavailable to assist with physical restraint when needed (eTable 3, Appendix). Symptoms of post-traumatic stress after the incident, such as repetitive thoughts about the event or severe anxiety were reported to be present by 541 (88.4%) victims, and nearly half (n = 303; 49.5%) considered leaving their job following the assault. A total of 61 workers (10.0%) required time off work following a violent incident, whether for a temporary period or permanently. Of these, seven cases (11.5%) resulted in permanent leave. Workplace performance was reported as negatively impacted in 460 (75.2%) cases, and 458 (74.8%) respondents reported changes in their interactions with patients after the violence (Table 2).

More than half of staff victims (n = 313; 51.1%) did not report the incident to their supervisor. Reasons for not reporting included perceptions that doing so would be ineffective (n = 159, 50.8%), lack of time (n = 94, 30.0%), fear of negative consequences (n = 67, 21.4%), or uncertainty about the reporting process (n = 63, 20.1%; Table 2).

Less than one-third of respondents (n = 227, 29.5%) indicated having an established institutional protocol for managing agitation. The most commonly chosen treatment for severe agitation by clinicians was the combination of haloperidol and promethazine, accounting for 34.5% (n = 265) of responses. This was followed by the combination of haloperidol and a benzodiazepine at 26.5% (n = 204). A benzodiazepine used alone was reported by 107 respondents (13.9%), while haloperidol was used by 91 reapondents (11.8%), and ketamine by 80 (10.4%; eTable 3, Appendix 1).

## DISCUSSION

Our study highlights the high prevalence and impact of WPV among ED staff in Brazil, with 79.6% indicating exposure to some form of violence, including 79.5% verbal abuse and 12.1% physical assault. Additionally, 40.3% witnessed physical assault against co-workers. Workplace violence had consequences, with most victims reporting post-traumatic stress symptoms, nearly half considering leaving their job, and over 75.0% experiencing a negative impact on workplace performance and patient interactions. Despite these effects, more than half of the incidents were not formally reported, often due to perceptions of futility, time constraints, or fear of negative repercussions.

Our findings on the prevalence of overall and verbal abuse closely align with data of ED surveys in the United States, which indicate that 71.5–86.0% of the workers indicated verbal abuse, while 21.0–37.0% indicated being victims of physical assault.^6^,^9^–^11^ A systematic review and meta-analysis reported that 77.0% of ED staff experienced verbal violence, and 18.0% experienced physical abuse, with family members of patients being the most common perpetrators.^7^ In our study, although the prevalence of self-reported physical assault was low at 12.1%, a higher proportion of respondents, 40.3%, indicated witnessing physical assaults against coworkers. The lower incidence of self-reported physical assault compared to previous studies, along with the higher frequency of witnessed violence, may reflect a reduced perception of violence and higher social tolerance.^26^

Similar to other studies, we found that the most likely perpetrators of violence were family members and patients; however, up to 35.8% of the perpetrators were coworkers, which could influence the belief that WPV is an inherent part of the job, leading to the naturalization of violence. Violence by coworkers in the healthcare setting has been previously reported in several studies, mostly in the form of bullying.^27^,^28^ Differences in prevalence may be attributable to the time frame for assessing WPV, with most surveys evaluating a 12-month period 9,10, while our study examined the prior six months, similar to those conducted by McGuire et al.^6^,^11^ In comparison to primary healthcare settings, a systematic review by Tian et al reported that 33.8% of respondents experienced verbal abuse, while 8.5% indicated physical assault.^29^ The consistently higher rates of violence observed in EDs supports the hypothesis that these environments are at greater risk for WPV. This increased vulnerability may be attributed to factors such as the high volume of critically ill patients, crowding, and the emotionally charged interactions among patients, families, and healthcare workers during emergency care.

The prevalence of violence observed in our study may have been influenced by institutional factors, including the lack of protocols for managing severe agitation (reported by 70.5% of respondents), inadequate security staffing for managing physical restraint (74.6%), and insufficient staff training (96.6%). In the multivariable analysis, the absence of preventive measures established by the ED was associated with higher odds of violence. Additionally, the presence of an institutional protocol for managing severe agitation was identified as a protective factor in the univariable analysis but did not remain significant after multivariable analysis.

The absence of standardized institutional protocols likely contributes to suboptimal pharmacological management of severe agitation. This is evident in the reported treatment choices, with 34.5% of respondents favoring a combination of haloperidol and promethazine, 26.5% opting for haloperidol and benzodiazepines, and 10% using ketamine. Thus, a minority of staff select one of the last two medications as their first choice, which are level B and C recommendations by the American College of Emergency Physicians^30^ in the management of severe agitation. Structured de-escalation training for ED staff has been shown to positively affect the reporting of WPV incidents and possibly reduce its impact.^11^

The impact of WPV is evident in several key outcomes: only 9.4% of respondents reported feeling extremely or very safe during clinical work in the ED, and 88.4% of victims experienced post-traumatic stress symptoms. Additionally, our findings suggest that older age may serve as a protective factor, although years of experience did not demonstrate a significant association, consistent with previous studies.^31^,^32^ The age-related difference may be attributed to enhanced communication skills and behavioral strategies developed with age, as well as the increased vulnerability of less experienced young workers.^32^ Our study population consisted of younger respondents, with a mean age of 32 years. Notably, only 45.9% of respondents reported having more than five years of work experience, a proportion substantially lower than prior studies, which reported 68.6% to 72.2% of staff with similar levels of experience.^6^,^11^ This difference may be attributed to EM being a relatively recent specialty in Brazil and may reflect younger workers’ interest in study participation.^21^

A cross-sectional study conducted in a large urban ED in 2023 found that healthcare workers experienced WPV once every 3.7 shifts, with nurses and younger staff being at higher risk.^27^ Additionally, a descriptive study in an urban ED highlighted that WPV incidents occurred almost daily, with 20.0% involving physical violence and a significant portion involving racist, sexist, or homophobic bias.^33^ Our study found no significant difference in the risk of WPV between cisgender women and cisgender men. These findings are consistent with some studies.^6^,^10^ However, a study by Kowalenko et al^9^ reported that female emergency physicians were at a higher risk of experiencing physical assault, or being bullied by coworkers.^34^

## LIMITATIONS

Limitations of our study include that the sample was majorly represented by physicians, probably due to the distribution by ABRAMEDE’s mailing list, which is composed mainly of these healthcare workers. Therefore, our results may have limited generalizability; the sample of other workers such as nurses and nursing technicians who may be at higher risk of WPV was significantly smaller,.^27^

Furthermore, as it was a self-reported survey from the prior six months, there may have been recall bias and possibly those that had experienced violence were more likely to complete the questionnaire, which could lead to selection bias. Also, the length of the survey may have contributed to the high rate of incomplete responses.

Additionally, due to the subjective nature of the questions the survey was susceptible to different interpretations among the respondents even with the definitions provided in the instructions for each question. Finally, only 19.1% of the initial respondents screened answered the survey, which limits the generalizability of our study. We assessed the lower response rate by examining the characteristics of non-respondents, which closely resembled those of our sample. Additionally, we calculated the nonresponse bias, as recommended by the International Association for Health Professions Education guidelines. Despite the low response rate, the wave analysis revealed minimal differences between the responses.^25^, ^35^.

## CONCLUSION

Our study identified a high self-reported prevalence of workplace violence among healthcare Workers in the emergency department, emphasizing the importance of public and institutional awareness to create protocols aimed at the prevention, education and management of WPV. These findings suggest potential implications for healthcare workers’ mental health, workplace well-being, workforce attrition, and patient care.

## Supplementary Information



## Figures and Tables

**Table 1:** Demographic and work environment characteristics of survey respondents regarding workplace violence in Brazilian emergency departments.

Demographic	All respondents (N = 769)	No violence (N = 157)	Any volence (N = 612)	P-value
Age, years	32 (28, 39)	36 (29, 44)	31 (28, 38)	< .001
21–25	55 (7.2%)	15 (9.6%)	40 (6.5%)	
26–30	272 (35.4%)	35 (22.3%)	237 (38.7%)	
31–35	160 (20.8%)	27 (17.2%)	133 (21.7%)	
36–40	115 (15.0%)	27 (17.2%)	88 (14.4%)	
41–45	79 (10.3%)	19 (12.1%)	60 (9.8%)	
46 or older	88 (11.4%)	34 (21.7%)	54 (8.8%)	
Sex and Gender, n (%)				.01
Cisgender female	437 (56.8%)	80 (51.0%)	357 (58.3%)	
Cisgender male	318 (41.4%)	73 (46.5%)	245 (40.0%)	
Transgender female	0 (0.0%)	0 (0.0%)	0 (0.0%)	
Transgender male	3 (0.4%)	0 (0.0%)	3 (0.5%)	
Other gender	3 (0.4%)	3 (1.9%)	0 (0.0%)	
Did not disclose	8 (1.0%)	1 (0.6%)	7 (1.1%)	
Sexual orientation, n (%)				.77
Heterosexual	642 (83.5%)	136 (86.6%)	506 (82.7%)	
Homosexual	63 (8.2%)	11 (7.0%)	52 (8.5%)	
Bisexual	53 (6.9%)	8 (5.1%)	45 (7.4%)	
Pansexual	1 (0.1%)	0 (0.0%)	1 (0.2%)	
Other orientation	0 (0.0%)	0 (0.0%)	0 (0.0%)	
Did not disclose	10 (1.3%)	2 (1.3%)	8 (1.3%)	
Self-reported Race/skin-color, n (%)				.02
White	590 (76.7%)	118 (75.2%)	472 (77.1%)	
Black	24 (3.1%)	12 (7.6%)	12 (2.0%)	
Asian	8 (1.0%)	1 (0.6%)	7 (1.1%)	
Pardo	139 (18.1%)	26 (16.6%)	113 (18.5%)	
Other race	2 (0.3%)	0 (0%)	2 (0.3%)	
Did not disclose	6 (0.8%)	0 (0%)	6 (1.0%)	
Role, n (%)				< .001
Attending physician	520 (67.6%)	97 (61.8%)	423 (69.1%)	
Resident physician	126 (16.4%)	24 (15.3%)	102 (16.7%)	
Nurse	65 (8.5%)	11 (7.0%)	54 (8.8%)	
Respiratory therapist	16 (2.1%)	10 (6.3%)	(1.0%)	
Nursing technician	28 (3.6%)	9 (5.7%)	19 (3.1%)	
Other role	14 (1.8%)	6 (3.8%)	8 (1.3%)	
Years of experience, n (%)				< .001
Under 1 year	87 (11.3%)	21 (13.4%)	66 (10.8%)	
1–5 years	329 (42.8%)	49 (31.2%)	280 (45.8%)	
More than 5 years	353 (45.9%)	87 (55.4%)	266 (43.5%)	
Hospital region, n (%)				.02
Southeast	371 (48.2%)	78 (49.7%)	293 (47.9%)	
South	205 (26.7%)	35 (22.3%)	170 (27.8%)	
Northeast	120 (15.6%)	36 (22.9%)	84 (13.7%)	
West	51 (6.6%)	4 (2.5%)	47 (7.7%)	
North	22 (2.9%)	4 (2.5%)	18 (2.9%)	
Main place of work, n (%)				.07
Community ED	234 (30.4%)	33 (21.0%)	201 (32.8%)	
Small hospital (5–50 beds)	37 (4.8%)	8 (5.1%)	29 (4.7%)	
Medium hospital (51–150 beds)	93 (12.1%)	20 (12.7%)	73 (11.9%)	
Large hospital (>150 beds)	369 (48.0%)	87 (55.4%)	282 (46.1%)	
Other	36 (4.7%)	9 (5.7%)	27 (4.4%)	
ED population				.61
All populations	336 (43.7%)	71 (45.2%)	265 (43.3%)	
Adult - non trauma	353 (45.9%)	69 (43.9%)	284 (46.4%)	
Trauma	19 (2.5%)	6 (3.8%)	13 (2.1%)	
Pediatric	38 (4.9%)	7 (4.5%)	31 (5.1%)	
Unknown/not reported	23 (3.0%)	4 (2.5%)	19 (3.1%)	
Type of ED, n (%)				< .001
Public ED	525 (68.3%)	92 (58.6%)	433 (70.8%)	
Referral-only public ED	129 (16.8%)	37 (23.6%)	92 (15.0%)	
Private ED	92 (12.0%)	24 (15.3%)	68 (11.1%)	
Unknown/not reported	23 (3.0%)	4 (2.5%)	19 (3.1%)	

*ED*, emergency department.

**Table 2:** Prevalence of self-reported verbal or physical assault in the past six months, report of violence, and its impact on staff in Brazilian emergency departments.

Prevalence of workplace violence	N (%)
Any form of workplace violence	612 (79.6%)
Verbal abuse	611 (79.5%)
Threatening tone	596 (77.5%)
Abusive tone	575 (74.8%)
Verbal harrassment	343 (44.6%)
Other physical assault	211 (27.4%)
Physical assault	93 (12.1%)
With an object	24 (3.1%)
With body fluids	39 (5.1%)
Physical harm	83 (10.8%)
Sexual assult	11 (1.4%)
Other physical assault	11 (1.4%)
Who was the perpetrator of the violence	
Patient	494 (64.2%)
Family member	522 (67.9%)
Coworker	275 (35.8%)
Other perpetrator	32 (4.2%)
Was the attacker intoxicated by a substance? [N=612]	
Yes	118 (19.3%)
Did the attacker have an acute change in mental status? [N=612]	
Yes	127 (20.8%)
Reporting Workplace Violence	
Did you report the incident to your supervisor? [N=612]	
Yes	299 (48.9%)
No	313 (51.1%)
If you didn’t report the incident, why not? [N = 313]	
It wasn’t important	46 (14.7%)
Feeling of guilt or shame	22 (7.0%)
Fear of negative consequences	67 (21.4%)
Didn’t have time to report violence	94 (30.0%)
Reporting violence is useless	159 (50.8%)
It’s part of the job	46 (14.7%)
Didn’t know how to report violence	63 (20.1%)
Other reason	14 (4.5%)
Impact of Workplace Violence	
Do you believe being a victim of violence impacted your performance at work? [N = 612]	
No	152 (24.8%)
Yes- for the remainder of the shift	133 (21.7%)
Yes- for 1 day	18 (2.9%)
Yes-for 2–7 days	95 (15.5%)
Yes- for 2–3 weeks	61 (10.0%)
Yes- for 1–4 months	49 (8.0%)
Yes- for 2–6 months	104 (17.0%)
Do you believe being a victim of violence changed the way you interact with patients? [N = 612]	
Yes	458 (74.8%)
After the attack, did you experience repetitive thoughts, significant anxiety, loss of interest in daily activities, distance yourself from others, or avoid thinking about the incident? [N = 612]	
Never	71 (11.6%)
Rarely	126 (20.6%)
Sometimes	193 (31.5%)
Often	134 (21.9%)
Very often	88 (14.4%)
After the attack, did you take time off work? [N = 612]	
No	551 (90.0%)
Yes- for 1 day	7 (1.1%)
Yes- for 2–3 days	8 (1.3%)
Yes- for 1 week	13 (2.1%)
Yes- for 2–3 weeks	5 (0.8%)
Yes- for 1 month	6 (1.0%)
Yes-for 2–6 months	10 (1.6%)
Yes- for >6 months	5 (0.8%)
Yes- permanently	7 (1.1%)
Workplace Violence Against Colleagues	
In the past six months, have you witnessed any physical assault against a colleague? [N = 769]	
Physical assault	310 (40.3%)
With an object	56 (7.3%)
With body fluids	95 (12.4%)
Physical harm	243 (31.6%)
Sexual assault	9 (1.2%)
Other physical assault	27 (3.5%)

**Table 3:** Respondents and institutional characteristics associated with workplace violence in Brazilian emergency departments.

Characteristic	Univariable		Age and gender adjusted		Multivariable	
		
	Odds ratio (95%CI)	P-value	Odds ratio (95%CI)	P-value	Odds ratio (95%CI)	P-value
Age, per 5 years	0.81 (0.74, 0.89)	< .001	---	---	0.72 (0.61, 0.84)	< .001
Sex/gender						
Male	Reference	---	---	---	Not used	---
Female	1.32 (0.92, 1.88)	.13	---	---	--	
Race						
White	Reference	---	Reference	---	Not used	---
Non-White	0.86 (0.57, 1.29)	.47	0.87 (0.57, 1.33)	.52	--	
Sexual orientation						
Heterosexual	Reference	---	Reference	---	Not used	---
Not heterosexual	1.39 (0.82, 2.35)	.22	1.14 (0.66, 1.95)	.64	--	
Years of experience						
>5 years	Reference	---	Reference	---	Reference	---
1–5 years	1.87 (1.27, 2.76)	< .001	1.17 (0.71, 1.91)	.54	0.84 (0.43, 1.64)	.61
<1 year	1.03 (0.59, 1.78)	.92	0.60 (0.32, 1.13)	.11	1.06 (0.35, 3.22)	.91
Role						
Attending physician	Reference	---	Reference	---	Reference	---
Nurse	1.19 (0.59, 2.43)	.63	1.55 (0.73, 3.31)	.25	1.25 (0.55, 2.86)	.59
Resident	0.92 (0.57, 1.49)	.73	0.64 (0.39, 1.07)	.09	0.79 (0.36, 1.71)	.54
Emergency department (ED) region						
Southeast	Reference	---	Reference	---	Reference	---
South	1.29 (0.83, 2.01)	.25	1.27 (0.81, 2.00)	.30	0.90 (0.49, 1.63)	.72
Northeast	0.62 (0.39, 0.99)	.04	0.53 (0.33, 0.85)	< .001	0.28 (0.15, 0.52)	< .001
West	3.13 (1.09, 8.95)	.03	3.03 (1.05, 8.76)	.04	1.84 (0.51, 6.66)	.35
North	1.20 (0.39, 3.64)	.75	1.38 (0.44, 4.33)	.58	1.03 (0.25, 4.27)	.96
Place of work						
Large hospital (>150 beds)	Reference	---	Reference	---	Reference	---
Hospital (5–150 beds)	1.12 (0.69, 1.82)	.64	1.04 (0.64, 1.71)	.86	1.60 (0.80, 3.20)	.18
Community ED	1.88 (1.21, 2.92)	< .001	1.73 (1.11, 2.71)	.01	1.49 (0.78, 2.87)	.23
Other	0.93 (0.42, 2.04)	.85	1.61 (0.66, 3.94)	.29	1.13 (0.41, 3.07)	.81
ED population						
All populations	Reference	---	Reference	---	Not used	---
Adult medical	1.10 (0.76, 1.60)	.61	1.05 (0.71, 1.53)	.82	--	
Pediatric or surgical clinic	0.91 (0.46, 1.78)	.78	1.23 (0.60, 2.52)	.57	--	
Type of ED						
Private or referral-only public ED	Reference	---	Reference	---	Reference	---
Public ED	1.79 (1.24, 2.60)	< .001	1.87 (1.28, 2.75)	< .001	1.41 (0.83, 2.40)	.20
Institutional preventive measures						
Severe agitation protocol	0.46 (0.31, 0.70)	< .001	0.51 (0.33, 0.78)	< .001	0.65 (0.39, 1.08)	.09
Availability of security personnel	0.68 (0.47, 0.98)	.03	0.61 (0.42, 0.89)	.01	0.73 (0.44, 1.20)	.22
Staff training on how to deal with potentially violent patients	0.69 (0.28, 1.66)	.40	0.86 (0.35, 2.15)	.75	Not used	
Staff training on difficult communications	0.70 (0.38, 1.29)	.25	0.66 (0.35, 1.24)	.20	Not used	
No preventative measures	2.49 (1.74, 3.56)	< .001	2.47 (1.71, 3.57)	< .001	1.88 (1.10, 3.21)	.02
