# Supplementary material for: Prevalence and Impact of Violence Against Healthcare Workers in Brazilian Emergency Departments: A National Survey
Source: West J Emerg Med. 2025 Oct 17;26(6):1769–80. doi: 10.5811/westjem.45138 (PMC12698140; doi:10.5811/westjem.45138)
Supplement: Supplementary file 1 [file wjem-26-1769-s001.docx]

**Appendix 1**

**eFigure 1.** Responses inclusion flowchart.


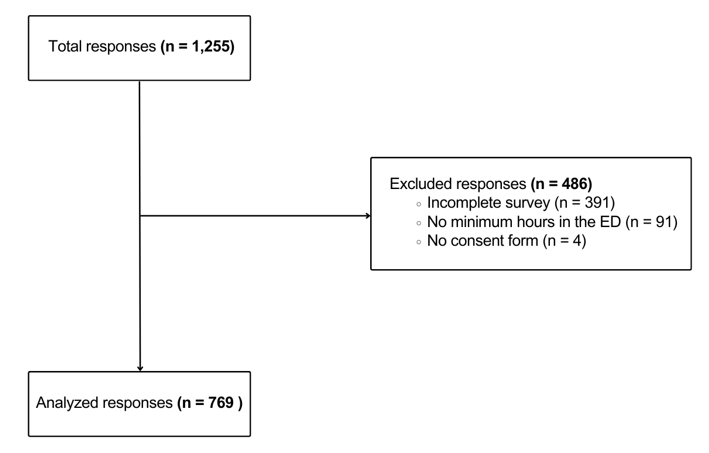


**eTable 1.** Frequency of type of workplace violence and their perpetrator in the past six months.

|  | **Never** | **One time** | **2-5 times** | **5-10 times** | **11 or more times** |
| --- | --- | --- | --- | --- | --- |
| **Verbal abuse** |  |  |  |  |  |
| Threatening tone | 173 (22.5%) | 68 (8.8%) | 254 (33.0%) | 146 (19.0%) | 128 (16.6%) |
| Abusive language | 194 (25.2%) | 102 (13.3%) | 226 (29.4%) | 136 (17.7%) | 111 (14.4%) |
| Verbal harassment | 426 (55.4%) | 67 (8.7%) | 139 (18.1%) | 71 (9.2%) | 66 (8.6%) |
| Other | 558 (72.6%) | 32 (4.2%) | 81 (10.5%) | 51 (6.6%) | 47 (6.1%) |
| **Physical assault** |  |  |  |  |  |
| With objects | 745 (96.9%) | 16 (2.1%) | 6 (0.8%) | 0 (0%) | 2 (0.3%) |
| With body fluids | 730 (94.9%) | 11 (1.4%) | 23 (3.0%) | 3 (0.4%) | 2 (0.3%) |
| Physical harm | 686 (89.2%) | 45 (5.9%) | 29 (3.8%) | 3 (0.4%) | 6 (0.8%) |
| Sexual assault | 758 (98.6%) | 6 (0.8%) | 3 (0.4%) | 1 (0.1%) | 1 (0.1%) |
| Other | 758 (98.6%) | 7 (0.9%) | 2 (0.3%) | 0 (0%) | 2 (0.3%) |
| **Perpetrator** |  |  |  |  |  |
| Patient | 275 (35.8%) | 113 (14.7%) | 244 (31.7%) | 76 (9.9%) | 61 (7.9%) |
| Family member | 247 (32.1%) | 85 (11.1%) | 229 (29.8%) | 132 (17.2%) | 76 (9.9%) |
| Coworker | 494 (64.2%) | 112 (14.6%) | 115 (15.0%) | 29 (3.8%) | 19 (2.5%) |
| Other | 737 (95.8%) | 11 (1.4%) | 10 (1.3%) | 3 (0.4%) | 8 (1.0%) |

**eTable 2.** Perceptions about workplace violence in the Emergency Department.

|  | **N (%)** |
| --- | --- |
| **Why do you think violence in the Emergency Department often goes unreported?** [N = 769] |  |
| Fear of negative consequences | 335 (43.6%) |
| Feelings of shame or guilt | 94 (12.2%) |
| Lack of time during the shift | 374 (48.6%) |
| Reporting violence is useless | 470 (61.1%) |
| Violence is part of the job | 159 (20.7%) |
| Lack of incentive or encouragement | 314 (40.8%) |
| Not knowing how to report | 256 (33.3%) |
| Other reason | 10 (1.3%) |
| **What is the main precipitating factor that leads to violence in the Emergency Department?** [N = 662] |  |
| Influence of alcohol or drugs | 65 (9.8%) |
| Mental disorder | 44 (6.6%) |
| High patient volume | 283 (42.7%) |
| Long patient waiting times | 169 (25.5%) |
| Other factor | 101 (15.3%) |
| **What preventive safety measures do you believe would reduce workplace violence?** [N = 769] |  |
| Active security team within the department | 535 (69.6%) |
| Metal detectors | 118 (15.3%) |
| Periodic staff training | 427 (55.5%) |
| Increased number of healthcare workers | 462 (60.1%) |
| Institutional protocols, such as in cases of severe agitation | 427 (55.5%) |
| Improving patient conditions, such as comfort and access  to food | 383 (49.8%) |
| Other preventive safety measure | 33 (4.3%) |
| **How safe do you feel in the Emergency Department?** [N = 769] |  |
| Extremely safe | 5 (0.7%) |
| Very safe | 67 (8.7%) |
| Moderately safe | 321 (41.7%) |
| Not very safe | 259 (33.7%) |
| Not safe | 117 (15.2%) |

**eTable 3.** Institutional resources available to avoid workplace violence.

|  | **N (%)** |
| --- | --- |
| **Are there established procedures for reporting violence in your workplace?** [N=769] |  |
| Yes | 249 (32.4%) |
| No | 273 (35.5%) |
| I don’t know | 247 (32.1%) |
| **Are you encouraged to report violence in your workplace?** [N=769] |  |
| Yes | 284 (36.9%) |
| No | 485 (63.1%) |
| **Are there any preventive measures against workplace violence in your institution?** [N=769] |  |
| None available | 496 (64.5%) |
| Protocol for cases of severe psychomotor agitation | 103 (13.4%) |
| Staff training on how to deal with potentially violent patients | 26 (3.4%) |
| Staff training on difficult communications | 57 (7.4%) |
| Presence of security staff | 195 (25.4%) |
| Presence of metal detectors | 0 (0%) |
| Other preventive measure | 4 (0.5%) |
| **If there is a need for physical containment in your institution, are there security workers available?** [N=769] |  |
| Yes | 300 (39.0%) |
| **Is there a protocol in your institution for cases of psychomotor agitation?** [N=769] |  |
| Yes | 227 (29.5%) |
| **In cases of severe psychomotor agitation, what pharmacological intervention would you choose?** [N=769] |  |
| Ketamine | 80 (10.4%) |
| Haloperidol | 91 (11.8%) |
| Benzodiazepine | 107 (13.9%) |
| Haloperidol and Benzodiazepine | 204 (26.5%) |
| Haloperidol and Promethazine | 265 (34.5%) |
| Propofol | 3 (0.4%) |
| Other medication | 19 (2.5%) |

**eTable 4.** Survey questions

| **I. Demographics** |
| --- |
| 1. Date of birth: ______ |
| 2. Gender  o Cisgender woman  o Transgender woman  o Cisgender man  o Transgender man  o Non-Binary  o Other  o I would rather not answer |
| 3. Sexual orientation  o Heterosexual  o Homosexual  o Bisexual  o Pansexual  o Asexual  o Other  o I would rather not answer |
| 4. Self-declared race  o White  o Black  o Pardo  o Asian  o Indigenous  o I would rather not answer |
| 5. Role in the ED  o Attending physician  o Resident physician  o Nurse  o Resident nurse  o Physiotherapist  o Resident physiotherapist  o Nursing technician  o Other |
| 6. Years of experience  o Less than 1 year  o Between 1 and 5 years  o More than 5 years |
| 7. Main state of the country in which work  o Acre (AC)  o Alagoas (AL)  o Amapá (AP)  o Amazonas (AM)  o Bahia (BA)  o Ceará (CE)  o Distrito Federal (DF)  o Espírito Santo (ES)  o Goiás (GO)  o Maranhão (MA)  o Mato Grosso (MT)  o Mato Grosso do Sul (MS)  o Minas Gerais (MG)  o Pará (PA)  o Paraíba (PB)  o Paraná (PR)  o Pernambuco (PE)  o Piauí (PI)  o Rio de Janeiro (RJ)  o Rio Grande do Norte (RN)  o Rio Grande do Sul (RS)  o Rondônia (RO)  o Roraima (RR)  o Santa Catarina (SC)  o São Paulo (SP)  o Sergipe (SE)  o Tocantins (TO) |
| 8. Main place of work in the Emergency department  o Community ED  o Small hospital (5 to 50 beds)  o Medium-sized hospital (51 to 150 beds)  o Large hospital (> 151 beds)  o Other |
| 9. If other, where? _______ |
| 10. What type of population does the department serve? (Note: Regarding your main place of work)  o Adult clinical emergency  o Surgical emergency  o Pediatric emergency  o General emergency (all types of population) |
| 11. What type of service? (Note: Regarding your main place of work)  o Public ED  o Referral-only public ED  o Private ED |
| 12. In the last 6 months, have you worked at least 12 hours a week in the Emergency department?  o Yes  o No |
| **II. Verbal abuse** |
| 13. Have you suffered any type of verbal aggression at work in the last 6 months?  (Note: Verbal aggression is defined as: any form of mistreatment, explicitly spoken or implied, that causes feelings of devaluation or humiliation, through words in a derogatory tone, threats, accusations or disrespectful expressions)  o Yes  o No |
| 14. What type of verbal aggression have you suffered in the last 6 months?   \|  \| Not once \| Just once \| 2 to 5 times \| 5 to 10 times \| > 10 times \| \| --- \| --- \| --- \| --- \| --- \| --- \| \| Threatening tone \|  \|  \|  \|  \|  \| \| Abusive language \|  \|  \|  \|  \|  \| \| Verbal harassment \|  \|  \|  \|  \|  \| \| Another verbal abuse \|  \|  \|  \|  \|  \| |
| 15. If other verbal aggression, what? _______ |
| 16. What was the type of verbal harassment?  o Racial  o Gender  o Sexual  o Other |
| 17. If other, what type? _______ |
| **III. Physical assault** |
| 18. Have you suffered any type of physical aggression at work in the last 6 months?  (Note: physical aggression is defined as: use of physical force that causes physical, sexual or psychological harm)  o Yes  o No |
| 19. What type of physical aggression have you suffered in the last 6 months?   \|  \| Not once \| Just once \| 2 to 5 times \| 5 to 10 times \| > 10 times \| \| --- \| --- \| --- \| --- \| --- \| --- \| \| Assault with an object (including hospital equipment) \|  \|  \|  \|  \|  \| \| Assault with body fluids (e.g. saliva, urine, feces, wound exudate, blood or spit) \|  \|  \|  \|  \|  \| \| Physical harm (aggression in the form of punching, biting, rough handling, scratching, kicking, pushing) \|  \|  \|  \|  \|  \| \| Sexual assault \|  \|  \|  \|  \|  \| \| Other type of physical assault \|  \|  \|  \|  \|  \| |
| 20. If other type of physical aggression, what? _______ |
| 21. By whom was the verbal and/or physical aggression carried out in the last 6 months?   \|  \| Not once \| Just once \| 2 to 5 times \| 5 to 10 times \| > 10 times \| \| --- \| --- \| --- \| --- \| --- \| --- \| \| Patient \|  \|  \|  \|  \|  \| \| Visitor/family \|  \|  \|  \|  \|  \| \| Coworker \|  \|  \|  \|  \|  \| \| Other \|  \|  \|  \|  \|  \| |
| 22. If other, who? _______ |
| 23. Did the aggressor appear to be intoxicated by some substance?  o Yes  o No |
| 24. Did the aggressor appear to have some acute change in mental status?  (Note: acute change in mental status defined as acute alteration of consciousness and/or cognition such as delirium, withdrawal syndrome, encephalopathy, encephalitis, psychic disorganization or psychotic crisis)  o Yes  o No |
| 25. Did all the incidents of aggression occur at your primary workplace?  o Yes  o No |
| 26. If no, where did it occur? ________ |
| 27. Did you report and/or inform your supervision? (Note: in the last 6 months)  o Yes  o No |
| 28. Why didn't you report? (Note: in the last 6 months)  □ It wasn't important  □ Feelings of shame or guilt  □ Fear of negative consequences  □ I don't have time to notify  □ I believe it is useless  □ I believe it is part of the job  □ I don't know how to notify  □ Other |
| 29. If other reason, what? ____ |
| 30. What time of day did it occur?  □ Morning  □ Afternoon  □ Night |
| **IV. Witnessed physical assault** |
| 31. Have you witnessed any type of physical assault at work against a colleague in the last 6 months? (Note: physical aggression is defined as: use of physical force that causes physical, sexual or psychological harm)  O Yes  O No |
| 32. What type of physical aggression? (Note: in the last 6 months)  □ Assault with an object (including hospital equipment)  □ Aggression with body fluids (e.g. saliva, urine, feces, blood, spit)  □ Physical harm (aggression in the form of punching, biting, rough handling, scratching, kicking, pushing)  □ Sexual assault  □ Other type of physical aggression |
| 33. If other type, what? _____ |
| 34. What was this person's role?  O Attending physician  O Resident physician  O Nurse  O Resident nurse  O Physiotherapist  O Resident physiotherapist  O Nursing technician  O Other |
| 35. If other, what? ____ |
| 36. Did this coworker take time away from work?  O Yes  O No  O I don't know |
| **V. Report and risk factors** |
| 37. Are there established procedures for reporting violence in your workplace?  O Yes  O No  O I don't know |
| 38. Are you encouraged to report violence in your workplace?  O Yes  O No |
| 39. Why do you think violence in the Emergency Department is usually unreported?  □ Fear of negative consequences  □ Feelings of shame or guilt  □ Lack of time to notify during the shift  □ Belief that is useless  □ Belief that it is part of the job  □ Lack of encouragement/encouragement  □ Not knowing how to notify  □ Other |
| 40. If other reason, what? ____ |
| 41. In your opinion, what was the main precipitating factor that led to the violence?  O Influence of alcohol/drugs  O Major mental disorder  O Number of patients  O Extended waiting time  O Other |
| 42. If another factor, which one? ____ |
| 43. Is there an institutional protocol for cases of psychomotor agitation?  O Yes  O No  O I don't know |
| 44. What measures do you believe would lead to an improvement in this problem?  □ Active security team  □ Metal detector  □ Periodic team training  □ Increase in the number of healthcare workers  □ Institutional protocols, such as in cases of severe agitation  □ Improvement of conditions for patients, such as greater comfort and access to food  □ Other |
| 45. If other measure, which one? ____ |
| 46. If there is a need for physical containment in your institution, are there security workers to assist in the process?  O Yes  O No  O I don't know |
| 47. In case of severe psychomotor agitation, what is your pharmacological measure of choice? (Note: severe agitation is defined as a score on the RASS scale + 4 (openly combative, violent and generating immediate danger to the themselves and others))  O Ketamine  O Haloperidol  O Benzodiazepine  O Haloperidol + benzodiazepine  O Haloperidol + promethazine  O Propofol  O Other |
| 48. If other measure, what? _____ |
| **VI. Violence impact** |
| 49. How safe do you feel in the Emergency Department?  O Extremely safe  O Very safe  O Moderately safe  O Unsafe  O Nothing safe |
| 50. Have you ever witnessed a patient and/or visitor carrying a fire gun during care?  O Yes  O No |
| 51. Do you believe that being a victim of aggression impacted your performance at work?  O Yes  O No |
| 52. If yes, for how long?  O The remainder of the shift  O 1 day  O 2 to 7 days  O 2 to 3 weeks  O 1 to 4 months  O For more than 5 months |
| 53. Do you believe that being a victim has changed the way you interact with patients?  O Yes  O No |
| 54. After the attack, did you experience repetitive thoughts about the incident, significant anxiety, distancing yourself from others, losing interest in daily activities or avoiding thinking about what happened?  O Very common  O Often  O Eventually  O Rarely  O Never |
| 55. Did you need to take time off from work after the aggression?  O Yes  O No |
| 56. How long were you away from work?  O 1 day  O 2 to 3 days  O 1 week  O 2 to 3 weeks  O 1 month  O 2 to 6 months  O More than 6 months  O Permanently |
| 57. Did you consider leaving your job after the incident?  O Yes  O No |
| 58. Was any action taken after the incident?  O Yes  O No |
| 59. Were there any consequences for the aggressor?  □ None  □ Verbal warning  □ Discontinuation of care  □ Reported to the police  □ Process opening  □ I don't know  □ Other |
| 60. If other, what? ____ |
| 61. Was any type of psychological support or institutional support offered after the incident?  O Yes  O No |
| 62. How satisfied were you with the way the incident was handled?  O Extremely satisfied  O Very satisfied  O Moderately satisfied  O Not very satisfied  O Very dissatisfied |
| 63. Are there any preventive measures in your institution? (Note: Select all the measurements that exist in your institution)  □ Does not exist  □ Institutional protocol for severe agitation  □ Training in how to deal with potentially violent patients  □ Difficult communications training  □ Presence of security staff  □ Metal detectors  □ Other |
| 64. If other, which one? ____ |
